# Healthcare utilization and chronic condition clusters in multimorbidity patients using weighted k-means: a register-based study in Denmark

**DOI:** 10.1007/s13755-026-00427-0

**Published:** 2026-01-15

**Authors:** Danny J. Anthonimuthu, Nikolaj N. Holm, Anders Stockmarr, Ann-Dorthe Zwisler, Flemming W. Udsen

**Affiliations:** 1 Department of Health Science and Technology, Faculty of Medicine, Aalborg University, Aalborg, Denmark; 2 Department of Applied Mathematics and Computer Science, Technical University of Denmark, Copenhagen, Denmark; 3 Department of Oncology, Clinic for Palliative Medicine and Rehabilitation, Rigshospitalet, Copenhagen, Denmark; 4 Department of Clinical Medicine, University of Copenhagen, Copenhagen, Denmark; 5 Department of Clinical Research, Copenhagen University Hospital Amager and Hvidovre, Copenhagen, Denmark; 6 Innovation and Research Centre for Multimorbidity, Slagelse Hospital, Slagelse, Denmark; 7 Department of Clinical Medicine, Danish Center for Health Services Research, Aalborg University, Aalborg, Denmark

**Keywords:** Multimorbidity, Health care utilization, Multiple long-term conditions, Machine learning, Artificial intelligence, Unsupervised learning, Clustering, k-means

## Abstract

**Background:**

The growing burden of multimorbidity challenges the healthcare system due to increased healthcare utilization and uncoordinated care. Identifying patients with multimorbidity who have high healthcare utilization is essential to improve management and reduce pressure on the healthcare system.

**Objective:**

This study aims to identify and characterize clusters of patients with multimorbidity based on both their chronic conditions and healthcare utilization patterns.

**Methods:**

A weighted K-means method was applied to a population of 1,184,334 individuals with two or more out of 33 chronic conditions, defined using diagnostic algorithms based on ICD-10 and ATC codes. Sociodemographic variables were applied to describe the identified clusters.

**Results:**

Four clusters were identified based on chronic conditions and healthcare utilization. Cluster 1 had the highest healthcare utilization and a high burden of both somatic and mental conditions, combined with low social status. Cluster 2, consisting primarily of younger women with mental conditions, showed high use of psychological services, and few somatic conditions. The largest cluster, cluster 3, had low healthcare utilization and consisted of individuals with common, manageable conditions, and relatively high social status. Cluster 4 was defined by older individuals with complex somatic conditions requiring frequent contact with general practitioners and specialists.

**Conclusion:**

The identified clusters showed varying chronic condition patterns and levels of healthcare utilization. The findings underscore the importance of tailored strategies, particularly for multimorbidity patients with mental conditions taking social status into account, in order to improve care and manage resource use more effectively.

## Introduction

Multimorbidity, defined as the co-occurrence of two or more chronic conditions, affects up to 37.2% of the global population, depending on study population and methodology, and is expected to rise due to an aging population [1, 2]. Multimorbidity is associated with increased healthcare utilization, including longer hospital stays, higher medication usage, fragmented care, and uncoordinated treatments [3, 4]. The annual cost per person with multimorbidity can range from I$800 to I$150,000, depending on the specific combination of conditions [5]. Thus, identifying patients with multimorbidity with high healthcare utilization is crucial for effective management and reducing strain on healthcare systems.

An approach to identifying these patients is patient segmentation, which involves grouping individuals into more homogenous categories based on, e.g., their health conditions. Patients with multimorbidity have different combinations of chronic conditions and care requirements, making it essential to identify and understand these differences to tailor treatment strategies [6]. Mesa‐Melgarejo et al. [7] demonstrated that a tailored case management model for patients with multimorbidity improved quality of life while reducing the burden on caregivers and healthcare [7].

However, patient segmentation is challenging due to the high heterogeneity in multimorbidity, which complicates the understanding of how specific combinations of chronic conditions influence health outcomes and healthcare utilization. Such complexity arises from not only the chronic conditions but also factors like sociodemographic characteristics, healthcare utilization patterns, and medication use, all of which interact in complex ways that can be difficult to gain a comprehensive overview and apply in clinical practice [8, 9].

One way to address these challenges is by applying advanced data analysis methods, such as cluster analysis, to uncover patterns that are not apparent through simpler analyses. Several studies have explored cluster analysis in multimorbidity, where they identify patterns labeled as metabolic and neurovascular clusters [10, 11] or patterns involving metabolic, musculoskeletal, and substance-related mental conditions [12]. Cluster analysis has also been applied in hospital populations to map chronic condition patterns and group patients with similar conditions [13].

In Denmark, several studies have investigated cluster analysis among patients with multimorbidity, focusing on the patterns of chronic conditions and their associations with healthcare utilization [14–18]. A common finding across the studies was that several clusters were associated with specific socioeconomic patterns, such as education level and income [14, 16, 18], as well as healthcare utilization patterns, including hospital admissions and medication use [14, 15, 17]. Furthermore, the studies primarily focused on a population-level approach, where patients were grouped based on chronic conditions and later profiled according to other variables, such as socioeconomic status or healthcare use. This approach may have certain limitations, as the most prevalent conditions in the population often dominate the clustering process, potentially overshadowing less common but more resource-intensive combinations of chronic conditions. Additionally, while this method captures interactions between chronic conditions, it does not account for potential interactions of sociodemographic factors or healthcare utilization within the clustering process itself.

Due to the differences in how certain chronic conditions are treated, it would be valuable to include information regarding healthcare utilization in the cluster analysis. This would not only help identify patients with similar chronic conditions but also those with comparable patterns of healthcare utilization.

The aim of this study is to identify patterns in patients with multimorbidity, based on combinations of chronic conditions, but also explicitly incorporating healthcare utilization into the clustering process, rather than applying it as a descriptive factor in later analyses. The results may support the development of targeted interventions to optimize healthcare utilization among individuals with multimorbidity.

## Methods

### Data foundation

The study population included all individuals, including both adults and children, with 2 or more chronic conditions among the 33 conditions outlined in Table 1. These individuals were living in Denmark as of January 1st 2019, and the total cohort consisted of 1,184,334 individuals. The data included 3 elements: presence status of the 33 chronic conditions (Table 1), utilization of healthcare services, and sociodemographic variables. Data were obtained from the Danish National Patient Register [19], the Danish Register of Causes of Death [20], the Danish National Prescription Registry [21], the Danish National Health Service Register [22], and the Danish Population Education Register [23].

**Table 1:** Chronic conditions and risk factors included in this study

Category	Chronic condition
Circulatory system	
	Ischemic heart disease
	Atrial fibrillation
	Heart failure
	Peripheral artery occlusive disease
	Hypertension (Risk factor)
	Stroke
	High cholesterol (Risk factor)
Endocrine system	
	Diabetes I
	Diabetes II
	Obesity (Risk factor)
Pulmonary system	
	COPD
	Allergies
Musculoskeletal system	
	Joint disease
	Osteoporosis
	Osteoarthritis
Cancers	
	Cancer of digestive organs
	Cancer of respiratory and intrathoracic organs
	Skin cancer
	Breast cancer
	Cancer of genital organs
	Other cancer excluding metastases
Neurological system	
	Epilepsy
	Parkinson’s disease
	Multiple sclerosis
Mental health conditions	
	Dementia
	Schizophrenia
	Depression
	Anxiety
	Addictive disorder
	Personality disorder
Urogenital system	
	Chronic kidney disease
Gastrointestinal system	
	Chronic liver disease
	Inflammatory bowel disease

Comprehensive factors, such as physical activity and nutrition, are not available in national registers. As the most suitable alternatives, risk factors were chosen to include aspects of lifestyle. The chronic conditions and risk factors included in this study were derived using diagnostic algorithms that were inspired by Prior et al. [24], Hvidberg et al. [25], and Schramm et al. [26]. These diagnostic algorithms were based on ICD-10 and ATC codes. A detailed description of the diagnostic algorithms is provided in Supplementary Material 1. The use of diagnostic algorithms captures patients treated in both primary and secondary care, including those receiving medication without hospitalization. To provide a more comprehensive representation of multimorbidity, a broad range of chronic conditions was included. The selected diagnoses reflect those commonly observed in the primary sector (see Table 1).

#### Healthcare utilization variables

The healthcare utilization variables included hospitalizations, bed days, medication usage, out-patient hospital visits, general practitioner visits, specialist doctor visits, and psychologist visits. All variables were accumulated for the year 2018. Hospitalizations and corresponding bed days included both psychiatric and somatic and were based on acute admissions. Medication usage reflected overall drug consumption by indicating the number of unique ATC code redeemed by each patient. The focus for general practitioner visits, specialist doctor visits, and psychologist visits was on physical consultations, defined based on medical specialty codes. Other contacts, such as transportation services, distance allowances, prescription renewals, telephone consultations, and email correspondence, were excluded from these variables. The included/excluded medical specialty codes can be seen in supplementary material 2.

#### Sociodemographic variables

The sociodemographic variables included age, sex, family type, region of residence, employment status, education, and income. Age was categorized into six intervals: < 16, 17–24, 25–44, 45–64, 65–84, and > 85 years of age. Family type was divided into five mutually exclusive categories: married couple, registered partnership, co-residing couple (not legally married or registered partners, but with at least one common child), cohabiting couple (unmarried, childless couples of opposite sexes with less than 15 years of age difference and no close family relation), and single. Each individual was assigned to one category only. The region variable included five categories representing the regions of Denmark: North Denmark Region, Central Denmark Region, Region of Southern Denmark, Capital Region of Denmark, and Region Zealand. Employment status was classified into seven categories: self-employed, employee, unemployed, in education, early retirement, retired, and receiving social assistance. Education was defined as the highest completed level of education and categorized into four groups based on the length of education: none (≤ 10 years), short (11–14 years), medium (14–17 years), and long (≥ 17 years). However, some individuals had missing data for employment status or education and were therefore placed in the "unknown" category for both variables. Finally, income was divided into quartiles (Q1-Q4) based on the distribution within the entire population.

### K-means clustering

The K-means method (Hartigan-Wong algorithm) was applied to cluster the data based on chronic condition and healthcare utilization variables. As a first step, all variables were standardized to have zero mean and unit variance. Since there were more chronic condition variables (n = 33) compared to healthcare utilization variables (n = 7), the chronic condition variables would naturally have a greater influence in the K-means clustering. To balance the contribution of chronic condition and healthcare utilization variables in the K-means algorithm, we scaled the healthcare utilization variables by $w=\sqrt{\frac{33}{7}}$. The ratio $\frac{33}{7}$ reflects the average ratio of their pairwise squared Euclidean distances under standardization. Since we are working with squared distances, the standardized healthcare utilization variables are multiplied by $\sqrt{\frac{33}{7}}$, which ensures equal contribution from both domains.

In order to find the most optimal number of clusters (k) for the dataset, we took an explorative approach. For each run, we used 25 initial random centroids and the solution with the lowest Within Cluster Sum of Squares (WCSS) was chosen, the WCSS was calculated based on the scaled input variables. For each k, the K-means algorithm was run 200 times, where the minimum WCSS was recorded. If the minimum value of WCSS appeared more than 10 times out of the 200 runs, the clustering with the minimum value of WCSS was used. Otherwise, an additional 100 runs were executed, after which the clustering with the lowest WCSS was used.

**Fig. 1:**
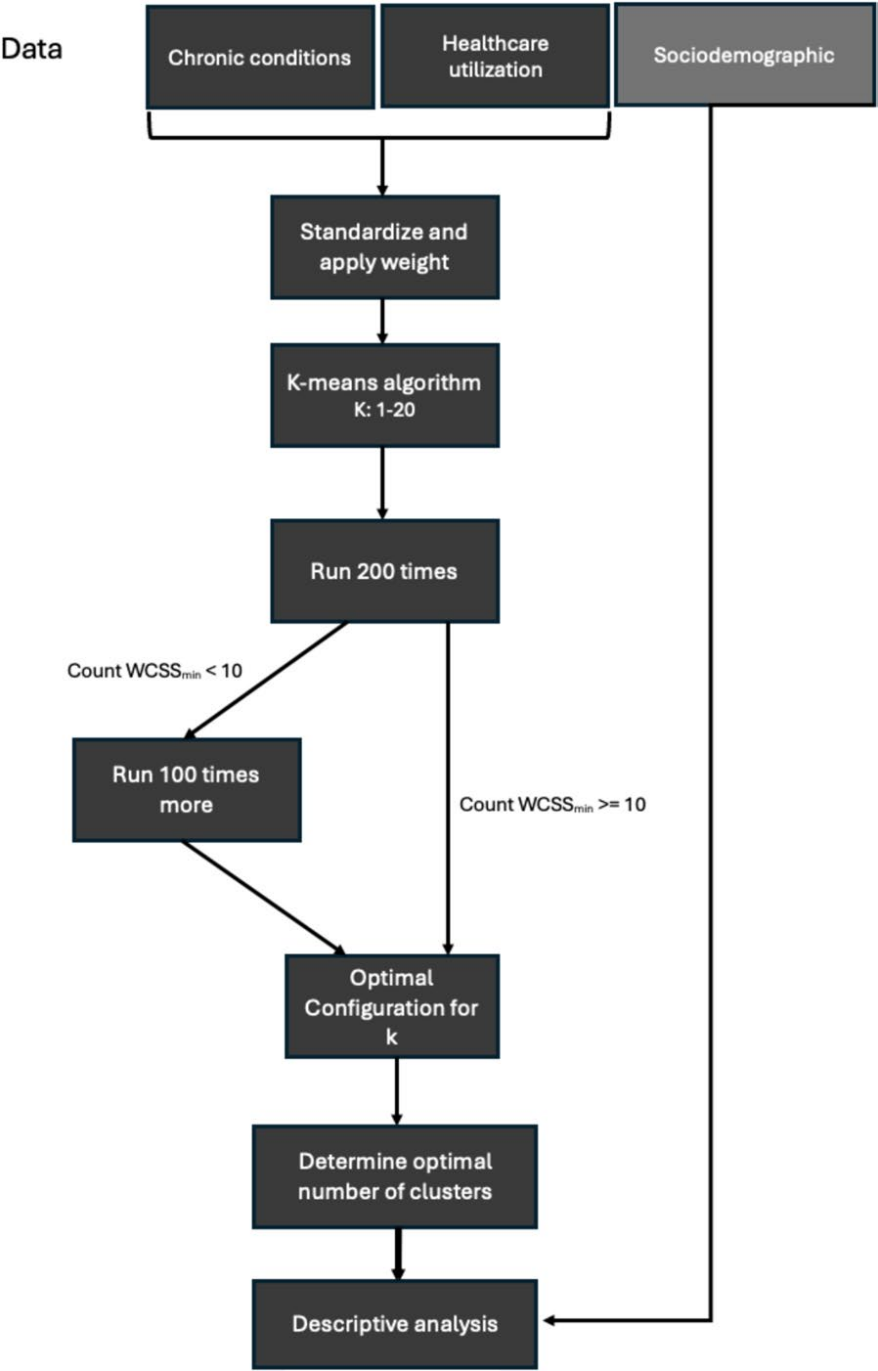
Overview of the analytic process, where it begins with the division of data into three segments. Hereafter, k-means clustering is performed on two data segments to group data into clusters. The optimal number of clusters (k) is determined using statistical evaluation methods, such as elbow method, silhouette score, and Calinski-Harabasz index. Finally, descriptive analysis is applied to examine the distribution of sociodemographic variables within the identified clusters

To determine the optimal number of clusters, models with the minimum WCSS for each k between 2 and 20 were visualized using an elbow plot, supplemented by the Calinski-Harabasz index [27] and a simplified silhouette score [28]. The whole process can be seen in Fig. 1.

### Descriptive analysis

To explore the relationships within the clusters, descriptive analysis was used. It offers insights into the distribution of sociodemographic variables within the clusters, including age, gender, education level, and income. Healthcare utilization variables were reported as means for each cluster, whereas sociodemographic variables were reported as the percentage distribution across their respective categories. In addition, the average age was also reported for each cluster. Examining these variables across the dataset and within each cluster helps identify notable patterns or outliers.

## Results

### Number of clusters

**Fig. 2:**
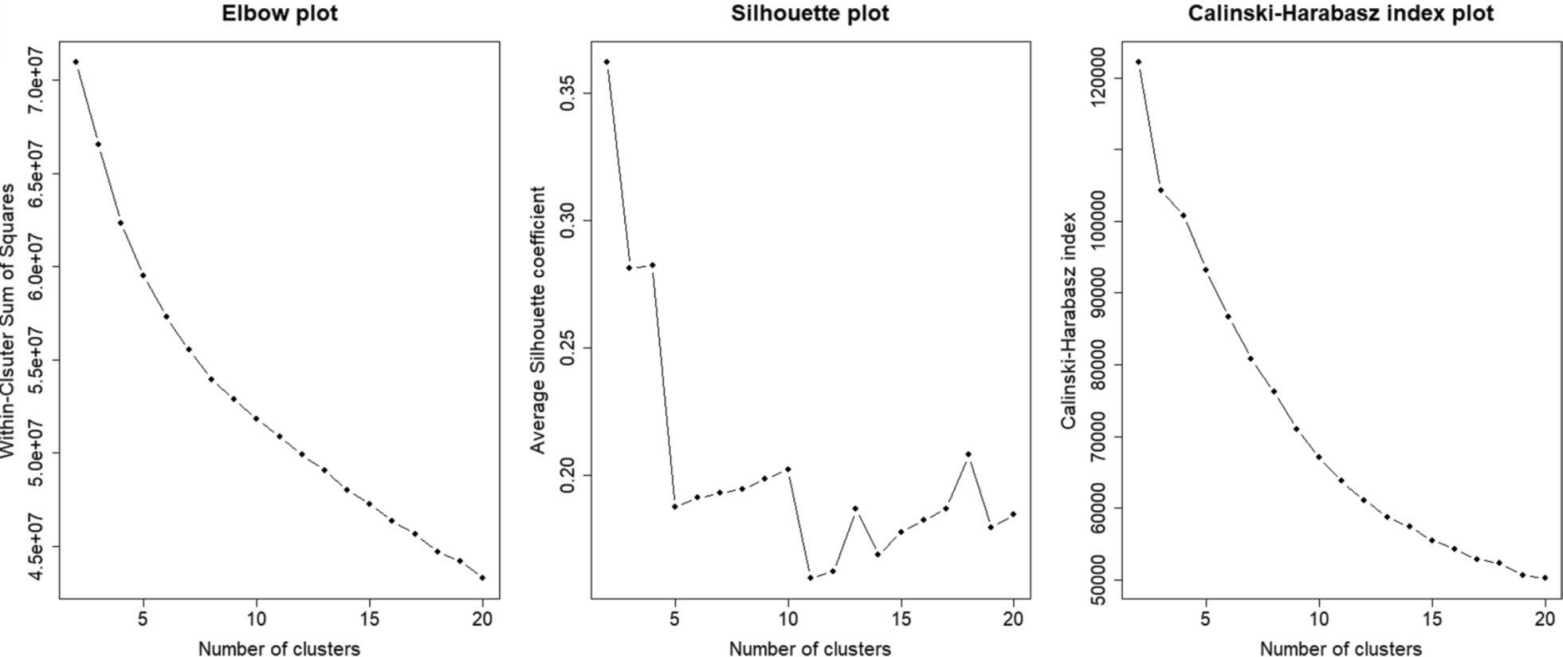
presents the elbow plot, silhouette score, and Calinski-Harabasz index. The number of clusters ranges from 2 to 20

The optimal number of clusters was determined using a combination of an elbow plot, silhouette score, and Calinski-Harabasz index. The corresponding plots are shown in Fig. 2.

As seen in the elbow plot in Fig. 2, there was no clear “elbow” indicating the optimal number of clusters. Additionally, WCSS decreased as the number of clusters increased. The decision was additionally supplemented by the silhouette score and the Calinski-Harabasz index. In the elbow plot, the presumed elbow region appeared to be between 4 and 7 clusters. Both the Calinski–Harabasz and Silhouette indices suggested a 2-cluster solution but also yielded acceptable values for 3 and 4 clusters. Considering statistical criteria, model simplicity, and clinical interpretability, 4 clusters were identified as the optimal choice.

### Characterization of clusters

In general, the average age of the multimorbid population was 65 years and included a slightly larger proportion of women (54.5%). On average, individuals in this population have 3.26 chronic conditions which can be seen in Table 2. As seen in Table 3, the three most prevalent chronic conditions were hypertension (70%), high cholesterol (45%), and osteoarthritis (23%).

**Table 2:** shows the prevalence of the sociodemographic variables for the four clusters, as well as for the entire multimorbidity population (All)

	Cluster 1	Cluster 2	Cluster 3	Cluster 4	All
Cluster size N, (%)	24,017 (2.03%)	5,035^a^ (0.43%)	918,901^b^ (77.59%)	236,381 (19.96%)	1,184,334 (100%)
Sociodemography					
Age (years) < 16	0.87%	0.26%^a^	0.92%^b^	0.32%	0.80%
Age (years) 17–24	1.92%	3.08%^b^	1.07%	0.61%^a^	1.0%
Age (years) 25–44	9.74%	26.77%^b^	7.70%	4.73%^a^	7.22%
Age (years) 45–64	26.2%	47.58%^b^	32.13%	22.24%^a^	30.1%
Age (years) 65–84	50.39%	21.83%^a^	49.78%	59.39%^b^	51.58%
Age (years) > 85	10.88%	0.5%^a^	8.41%	12.72%^b^	9.29%
Age (years) all (mean)	64.81	51.52^a^	64.43	69.16^b^	65.33
Male	52.85%^b^	24.44%^a^	45.84%	43.84%	45.48%
Female	47.15%^a^	75.56%^b^	54.16%	56.16%	54.52%
Family type					
Registered partnership	0.1%^a^	0.2%^b^	0.14%	0.11%	0.13%
Cohabiting couple	2.47%	4.86%^b^	2.75%	1.92%^a^	2.6%
Co-residing couple	4.18%^a^	8.76%b	5.41%	4.71%	5.26%
Married couple	40.07%^a^	43.08%	52.09%^b^	50.67%	51.52%
Single	53.22%^b^	43.1%	39.62%^a^	42.59%	40.49%
Region					
North Denmark Region	8.68%^a^	12.69%^b^	11.37%	11.53%	11.35%
Central Denmark Region	18.95%^a^	23.65%^b^	23.09%	22.79%	22.95%
Region of South Denmark	23.49%^a^	24.56%	23.91%	24.91%^b^	24.10%
Capital Region of Denmark	32.19%^b^	23.85%^a^	25.72%	25.72%	25.84%
Region Zealand	16.7%^b^	15.25%	15.9%	15.05%^a^	15.74%
Employment status					
Self-employed	1.4%^a^	2.74%^b^	2.39%	1.56%	2.21%
Employee	12.66%^a^	42.36%^b^	27.7%	15.19%	24.96%
Unemployed	1.93%	5.69%^b^	1.2%	1.05%^a^	1.20%
In education	1.12%	4.4%^b^	1.24%	0.64%^a^	1.13%
Early retirement	15.4%^b^	10.46%	7.7%^a^	8.24%	7.99%
Retired	57.41%	35.42%^a^	53.2%	67.77%^b^	56.13%
Social assistance	8.2%	13.05%^b^	4.67%	4.57%^a^	4.75%
Unknown/other	1.88%	1.97%^b^	1.91%	0.95%^a^	1.72%
Education					
None	29.65%	9.05%^a^	24.81%	30.38%^b^	25.95%
Short	36.39%^a^	44.52%^b^	38.39%	36.56%	38.0%
Medium	18.95%	16.64%^a^	19.45%^b^	18.47%	19.22%
Long	11.65%^a^	28.57%^b^	14.65%	12.33%	14.27%
Unknown	3.36%^b^	1.21%^a^	2.7%	2.27%	2.62%
Income (quartiles)					
Q1 (< 25%)	25.29%^b^	21.03%^a^	22.66%	25.08%	23.17%
Q2 (25–50%)	53.01%^b^	44.88%^a^	46.77%	50.54%	47.65%
Q3 (50–75%)	8.81%^a^	14.44%^b^	11.58%	10.32%	11.29%
Q4 (> 75%)	12.87%^a^	19.64%^b^	18.99%	14.06%	17.89%
Health service utilization (mean)					
Medication usage	9.30^b^	5.97	5.57 ^a^	8.09	6.15
Hospitalizations	4.15^b^	0.40	0.048^a^	0.52	0.23
Bed days	29.49^b^	1.67	0.14^a^	1.61	1.03
General practitioner	21.74	16.59	3.19^a^	23.10^b^	7.59
Specialist doctor	2.46	4.33^b^	0.46^a^	6.08	1.64
Psychologist	0.039	8.84^b^	0.0062^a^	0.024	0.048
Outpatient visits	16.13^b^	3.55	0.47^a^	3.64	1.43
Number of conditions					
2	20.14%^a^	50.58%^b^	45.28%	24.91%	40.73%
3	19.16%^a^	24.27%	26.59%^b^	24.78%	26.06%
4	17.11%	12.69%^a^	14.43%	20.54%^b^	15.70%
5	15.02%^b^	6.18%^a^	7.33%	13.84%	8.78%
6	10.67%^b^	3.51%^a^	3.52%	8.16%	4.59%
7	7.55%^b^	1.43%^a^	1.63%	4.22%	2.27%
8	4.81%^b^	0.87%	0.73%^a^	2.02%	1.07%
9	2.91%^b^	0.22%^a^	0.3%	0.91%	0.47%
≥ 10	2.55%^b^	0.26%	0.18%^a^	0.62%	0.32%
Number of conditions (mean)	4.51^b^	2.98^a^	3.08	3.85	3.26

^a^indicates the lowest value across clusters, whereas ^b^ indicates the highest value

**Table 3:** Shows the prevalence of risk factors and chronic conditions in the four clusters, as well as for the entire multimorbidity population (All)

Chronic condition	Cluster 1 (O/E)	Cluster 2 (O/E)	Cluster 3 (O/E)	Cluster 4 (O/E)	All
Ischemic heart disease	24.60% (1.47)^x^	10.54% (0.63)	15.21% (0.91)	21.94% (1.31)^x^	16.72%
Atrial fibrillation	22.78% (1.95)^x^	5.14% (0.44)	9.87% (0.85)	17.65% (1.51)^x^	11.66%
Heart failure	14.79% (2.75)^x^	2.56% (0.48)	4.51% (0.84)	7.83% (1.46)^x^	5.37%
Peripheral artery occlusive disease	14.03% (2.11)^x^	3.08% (0.46)	5.89% (0.88)	8.98% (1.35)^x^	6.66%
Hypertension	70.70% (1.01)^x^	46.07% (0.66)	68.11% (0.97)	78.56% (1.12)^x^	70.15%
Stroke	17.90% (1.98)^x^	5.72% (0.63)	8.49% (0.94)	10.36% (1.15)^x^	9.04%
High cholesterol	37.71% (0.83)	25.10% (0.55)	43.88% (0.97)	51.78% (1.14)^x^	45.25%
Diabetes I	9.18% (2.11)^x^	3.81% (0.88)	4.04% (0.93)	5.07% (1.17)^x^	4.35%
Diabetes II	21.10% (1.15)^x^	10.74% (0.59)	16.53% (0.90)	24.99% (1.37)^x^	18.29%
Obesity	12.64% (1.19)^x^	19.62% (1.85)^x^	10.37% (0.98)	11.16% (1.051)^x^	10.61%
COPD	22.09% (1.21)^x^	19.18% (1.05)^x^	17.06% (0.93)	22.80% (1.25)^x^	18.31%
Allergies	9.08% (0.71)	20.35% (1.59)^x^	12.39% (0.97)	14.76% (1.15)^x^	12.83%
Joint disease	2.98% (1.11)^x^	2.30% (0.86)	2.57% (0.96)	3.12% (1.16)^x^	2.69%
Osteoporosis	17.15% (1.14)^x^	11.64% (0.77)	14.19% (0.94)	18.12% (1.21)^x^	15.02%
Osteoarthritis	21.65% (0.94)	17.97% (0.78)	22.08% (0.96)	27.29% (1.18)^x^	23.10%
Cancer digestive	8.39% (4.06)^x^	1.53% (0.74)	1.86% (0.90)	2.25% (1.09)^x^	2.07%
Cancer respiratory	5.03% (5.21)^x^	0.66% (0.68)	0.79% (0.81)	1.25% (1.30)^x^	0.96%
Cancer skin	0.99% (1.20)^x^	0.87% (1.06)^x^	0.80% (0.98)	0.89% (1.07)^x^	0.84%
Cancer breast	4.07% (1.42)^x^	4.31% (1.50)^x^	2.83% (0.99)	2.82% (0.99)	2.86%
Cancer genital	5.86% (2.10)^x^	1.59% (0.57)	2.61% (0.94)	3.18% (1.14)^x^	2.79%
Cancer other	11.52% (4.04)^x^	2.20% (0.77)	2.57% (0.90)	3.11% (1.09)^x^	2.85%
Epilepsy	13.54% (2.25)^x^	10.15% (1.69)^x^	5.81% (0.97)	5.98% (0.99)	6.02%
Parkinson disease	1.82% (2.38)^x^	0.36% (0.47)	0.66% (0.87)	1.06% (1.39)^x^	0.77%
Multiple sclerosis	1.13% (1.37)^x^	1.45% (1.76)^x^	0.85% (1.03)^x^	0.68% (0.82)	0.82%
Dementia	3.56% (1.26)^x^	0.62% (0.22)	2.78% (0.98)	2.98% (1.05)	2.83%
Schizophrenia	4.35% (7.41)^x^	0.54% (0.91)	0.50% (0.86)	0.53% (0.90)	0.59%
Depression	33.14% (1.61)^x^	53.69% (2.61)^x^	19.34% (0.94)	23.26% (1.13)^x^	20.55%
Anxiety	4.54% (4.34)^x^	5.10% (4.88)^x^	0.94% (0.90)	1.01% (0.96)	1.05%
Addictive disorder	7.15% (6.27)^x^	1.05% (0.92)	1.03% (0.90)	0.98% (0.86)	1.14%
Personality disorder	2.00% (6.30)^x^	0.75% (2.37)^x^	0.28% (0.89)	0.27% (0.87)	0.32%
Kidney disease	11.07% (3.67)^x^	1.65% (0.55)	2.63% (0.87)	3.74% (1.24)^x^	3.02%
Liver disease	6.00% (3.35)^x^	1.33% (0.74)	1.72% (0.96)	1.63% (0.91)	1.79%
Bowel disease	8.35% (1.79)^x^	6.61% (1.42)^x^	4.50 (0.96)	4.92% (1.05)^x^	4.67%

For the four clusters, the observed/expected ratio (O/E) is provided in parentheses. If the O/E ratio is greater than 1, it is indicated with an ‘x’ in the table

#### Cluster 1

**Cluster size:** This cluster included 24,017 individuals, corresponding to 2.03% of the total multimorbid population, which was the second lowest in size compared to the other clusters.

**Utilization of healthcare services:** This cluster had the highest levels of medication usage (9.3), hospitalizations (4.2), bed days (29.5), and outpatient visits (16.1). General practitioner (21.7) and psychologist (0.039) consultations were the second highest, while specialist doctor visits ranked third among the clusters.

**Chronic conditions in the cluster:** This cluster had the highest average number of chronic conditions (4.5). The three most common conditions were hypertension (70.7%), high cholesterol (37.7%), and depression (33.1%), while the least prevalent were skin cancer (0.99%), multiple sclerosis (1.1%), and Parkinson’s disease (1.8%). Compared to the expected numbers in the multimorbid population, a higher observed occurrence was found for 30 out of the 33 chronic conditions. The greatest deviations were found for schizophrenia (O/E:7.41; corresponding to a 641% increase), personality disorder (6.30; 530%), and addictive disorder (6.27; 527%). Furthermore, it was worth noticing that kidney disease had a higher observed occurrence (3.67; 267%) compared to the other clusters. The remaining can be seen in Table 3.

**Combinations of chronic conditions in the cluster:** Among combinations of conditions in the chronic condition portfolios, hypertension, high cholesterol, and depression were the most prevalent (Table 4). When risk factors were excluded (Table 5 in appendix), depression remained a key component in most of the combinations of two and three, but it was less common in combinations of four. The combination of atrial fibrillation, heart failure, and ischemic heart disease was also frequent observed in the combinations of four chronic conditions.

**Sociodemographic characteristics:** The average age in this cluster was the third highest (64.8 years), and it had the highest proportion of men (52.9%). This cluster also had the highest rate of individuals living alone (53.2%) and the highest proportion of residents from Capital Region of Denmark (32.2%) among the clusters. It was also observed that proportion of residents in the North Denmark Region and Central Denmark Region was the lowest in this cluster. The percentage of retired individuals was the second highest (57.4%). Additionally, this cluster had the lowest level of higher educational attainment, with only 11.6% having a higher education degree. Incomes were the lowest in this cluster, with 78.3% falling within the first and second quartiles.

**Conclusion:** This cluster included individuals with hypertension, high cholesterol, addictive disorders, and especially mental conditions (depression, schizophrenia, and personality disorders). It had the highest average number of chronic conditions and the highest healthcare utilization. A large proportion of individuals in this cluster were single (53.2%). Additionally, this cluster ranked lowest in social position regarding education and income.

#### Cluster 2

**Cluster size:** The cluster consisted of the fewest individuals, N = 5,035, representing only 0.43% of the total population with multimorbidity.

**Utilization of healthcare services:** Psychologist consultations were the highest in this cluster (8.8), while bed days (1.67) and specialist doctor visits (4.33) were the second highest among the clusters. Additionally, medication usage (5.6), hospitalizations (0.05), general practitioner (3.2), and outpatient visits ranked (0.47) as the third highest.

**Chronic conditions in the cluster:** This cluster had the lowest average number of chronic conditions (2.98). Depression (53.7%), hypertension (46.1%), and high cholesterol (25.1%) were the most prevalent chronic conditions, while Parkinson Disease (0.36%), Schizophrenia (0.54%), and dementia (0.62%) were the least common. Furthermore, 11 of the 33 chronic conditions showed an observed/expected ratio above 1. The largest increases were observed for anxiety (O/E: 4.88; corresponding to a 388% increase), depression (2.61;161%), and personality disorder (2.37; 137%).

**Combinations of chronic conditions in the cluster:** In combination of two conditions, depression was prominent. In combination of three and four, depression, high cholesterol, and hypertension appeared in most combinations (Table 4). When risk factors were excluded ( Table 5 in appendix), depression played an even greater role, appearing in nearly every combination of chronic conditions. COPD and allergies also became more prominent. Additionally, osteoarthritis was more frequently observed in combinations of three and four.

**Sociodemographic characteristics:** This cluster had the lowest average age (51.5 years) among the four clusters. It had the highest proportion of women (75.6%). The majority were either single (43%) or married (43%), with the proportion of single individuals being the second highest and married individuals the third highest among the clusters. The largest share of individuals resided in region of south Denmark (24.6%), followed by Capital Region of Denmark (23.9%) and Central Denmark Region (23.7%). This cluster had the highest proportion of individuals in employment (42.4%), while the share of retired individuals was the lowest (35.4%). It also had the highest level of higher educational attainment (28.6%). Additionally, income was the highest, with 34.1% falling within the third and fourth quartiles.

**Conclusion:** This cluster consisted of the youngest individuals, with a high proportion of women (75.6%). It was characterized by mental disorders such as depression and anxiety, as well as hypertension and high cholesterol. While it had the lowest average number of chronic conditions, it had a high burden on psychologist visits. This cluster had the highest social position in terms of education and income.

#### Cluster 3

**Cluster size:** The cluster consisted of 918,901 individuals, accounting for 77.59% of all individuals with multimorbidity, making it the largest cluster.

**Utilization of healthcare services:** Medication usage (5.57), hospitalizations (0.048), bed days (0.14), general practitioner visits (3.19), specialist doctor visits (0.46), psychologist consultations (0.0062), and outpatient visits (0.47) were the lowest in this cluster.

**Chronic conditions in the cluster:** This cluster had the second lowest average number of chronic conditions (3.08). The three most prevalent chronic conditions were hypertension (68.11%), high cholesterol (43.88%), and osteoarthritis (22.08%), while the least prevalent were personality disorder (0.28%), schizophrenia (0.50%), and Parkinson Disease (0.66%). A higher prevalence than expected was only observed for multiple sclerosis (O/E:1.03, corresponding to a 3% increase).

**Combinations of chronic conditions in the cluster:** Hypertension and high cholesterol were prominent in combinations of two, three, and four chronic conditions (Table 4). When excluding the risk factors (Table 5 in appendix), osteoarthritis, COPD, and allergies emerged as the most prevalent conditions in these combinations.

**Sociodemographic characteristics:** The average age in this cluster was 64.4 years, making it the third highest among the clusters. A higher proportion of men (54.2%) than women was observed. The percentage of married individuals (52.1%) was the highest among the clusters, while the prevalence of single individuals was the lowest at 39.6%. Most individuals resided in the Capital Region of Denmark (25.7%), followed by the Region of Southern Denmark (23.9%) and the Central Denmark Region (23.1%). The proportion of retired individuals was the second lowest (53.2%), whereas the proportion of employed individuals was the second highest (27.7%) among the clusters. This cluster had the second-highest level of higher educational attainment (14.7%) and the second-highest income (30.6%) when combining the third and fourth quartiles.

**Conclusion:** This cluster included chronic conditions such as hypertension, high cholesterol, and osteoarthritis. It had a higher proportion of men (54.2%) than women. This cluster had the lowest utilization of healthcare services and the second-highest social position in terms of education and income.

#### Cluster 4

**Cluster size:** This cluster comprised 236,381 individuals, representing 19.96% of the multimorbid population and ranking as the second largest cluster.

**Utilization of healthcare services:** General practitioner (23.1) and specialist doctor (6.1) were the highest in this cluster. Additionally, medication usage (8.1), hospitalization (0.52), and outpatient visits (3.6) were the second highest, while bed days (1.61) and psychologist visits (0.024) were the second lowest among the clusters.

**Chronic conditions in the cluster:** The cluster had the second-highest average number of chronic conditions (3.85). Hypertension (78.56%), high cholesterol (51.78%), and osteoarthritis (27.29%) were considered the three most prevalent chronic conditions in the cluster, while personality disorder (0.27%), schizophrenia (0.53%), and multiple sclerosis (0.68%) were the three least prevalent. An observed/expected ratio above 1 was identified for 24 of the 33 chronic conditions. The most notable increases were observed for atrial fibrillation (O/E:1.51, corresponding to a 51% increase), heart failure (1.46; 46%), and Parkinson disease (1.39; 39%). The rest can be seen in Table 2.

**Combinations of chronic conditions in the cluster:** Hypertension was most prominent in combinations of two conditions, whereas both hypertension and high cholesterol were observed in all combinations of three and four (Table 4). When risk factors were excluded (Table 5 in appendix), osteoarthritis was the most prominent, followed by COPD and allergies.

**Sociodemographic characteristics:** The average age of the cluster is 69.2 years, which was the highest among the clusters. Additionally, a higher proportion of men (56.2%) than women was observed. In terms of family type, 50.7% were married, while 42.6% were single, which was the second-lowest among the clusters. Individuals in this cluster primarily resided in the Capital Region of Denmark (25.7%) followed by the Region of South Denmark (24.9%) and the Central Denmark Region (22.8%). The proportion of retired was 67.8%, which was the highest among the clusters. In contrast, individuals in employment were the second-lowest (15.19%). It was also noted that the cluster had the second-lowest level of higher educational attainment (12.33%). Income was also the second-lowest (24.4%), when combining the third and fourth quartiles.

**Conclusion:** This cluster included the oldest individuals, with a higher proportion of men (56.2%) than women. The individuals were primarily affected by hypertension, high cholesterol, and osteoarthritis. It had the highest proportion of retired individuals (67.8%). The cluster showed high healthcare utilization, particularly in terms of General Practitioner and specialist doctor visits. Additionally, it had the second-lowest social position based on education and income.

**Table 4:** presents the most prevalent combinations of two, three, and four chronic conditions (chronic condition portfolios) found within each cluster

2 conditions rank	Cluster 1	Cluster 1 Prevalence	Cluster 2	Cluster 2 Prevalence	Cluster 3	Cluster 3 Prevalence	Cluster 4	Cluster 4 Prevalence
1	Depression, Epilepsy	228 (0.95%)	Depression, Hypertension	211 (4.19%)	High cholesterol, Hypertension	65,141 (7.09%)	High cholesterol, Hypertension	7512 (3.18%)
2	Depression, Hypertension	147 (0.61%)	Allergies, Depression	179 (3.55%)	Hypertension, Osteoarthritis	26,767 (2.91%)	Hypertension, Osteoarthritis	4138 (1.75%)
3	Cancer other, Hypertension	131 (0.55%)	Depression, Obesity	162 (3.22%)	Allergies, COPD	21,013 (2.29%)	Atrial fibrillation, Hypertension	2725 (1.15%)
4	Atrial fibrillation, Hypertension	124 (0.52%)	Depression, Epilepsy	135 (2.68%)	Depression, Hypertension	15,090 (1.64%)	Allergies, COPD	2664 (1.13%)
5	Cancer digestive, Hypertension	123 (0.51%)	Allergies, COPD	115 (2.28%)	COPD, Hypertension	12,507 (1.36%)	Depression, Hypertension	2463 (1.04%)
3 conditions rank	Cluster 1	Cluster 1 Prevalence	Cluster 2	Cluster 2 Prevalence	Cluster 3	Cluster 3 Prevalence	Cluster 4	Cluster 4 Prevalence
1	High cholesterol, Hypertension, Ischemic heart disease	113 (0.47%)	Allergies, COPD, Depression	55 (1.09%)	Diabetes II, High cholesterol, Hypertension	24,068 (2.62%)	Diabetes II, High cholesterol, Hypertension	6010 (2.54%)
2	High cholesterol, Hypertension, Stroke	96 (0.4%)	Depression, High cholesterol, Hypertension	54 (1.07%)	High cholesterol, Hypertension, Ischemic heart disease	18,352 (2.00%)	High cholesterol, Hypertension, Ischemic heart disease	3184 (1.35%)
3	Depression, High cholesterol, Hypertension	59 (0.25%)	Depression, Hypertension, Obesity	32 (0.64%)	High cholesterol, Hypertension, Osteoarthritis	10,906 (1.19%)	High cholesterol, Hypertension, Osteoarthritis	2375 (1.00%)
4	Atrial fibrillation, Heart failure, Hypertension	54 (0.23%)	High cholesterol, Hypertension, Ischemic heart disease	32 (0.64%)	High cholesterol, Hypertension, Stroke	7692 (0.84%)	Atrial fibrillation, High cholesterol, Hypertension	1414 (0.60%)
5	Diabetes II, High cholesterol, Hypertension	48 (0.20%)	Diabetes II, High cholesterol, Hypertension	25 (0.50%)	Depression, High cholesterol, Hypertension	6098 (0.66%)	Depression, High cholesterol, Hypertension	1359 (0.57%)
4 conditions rank	Cluster 1	Cluster 1 Prevalence	Cluster 2	Cluster 2 Prevalence	Cluster 3	Cluster 3 Prevalence	Cluster 4	Cluster 4 Prevalence
1	Heart failure, High cholesterol, Hypertension, Ischemic heart disease	53 (0.22%)	Depression, High cholesterol, Hypertension, Ischemic heart disease	17 (0.34%)	Diabetes II, High cholesterol, Hypertension, Osteoarthritis	3649 (0.40%)	Diabetes II, High cholesterol, Hypertension, Osteoarthritis	1510 (0.64%)
2	Atrial fibrillation, High cholesterol, Hypertension, Ischemic heart disease	50 (0.21%)	Depression, Diabetes II, High cholesterol, Hypertension	16 (0.32%)	Diabetes II, High cholesterol, Hypertension, Ischemic heart disease	3515 (0.38%)	Diabetes II, High cholesterol, Hypertension, Ischemic heart disease	1448 (0.61%)
3	Depression, High cholesterol, Hypertension, Stroke	40 (0.17%)	Depression, High cholesterol, Hypertension, Osteoarthritis	14 (0.28%)	High cholesterol, Hypertension, Ischemic heart disease, Osteoarthritis	3195 (0.35%)	High cholesterol, Hypertension, Ischemic heart disease, Osteoarthritis	995 (0.42%)
4	Diabetes II, High cholesterol, Hypertension, Ischemic heart disease	39 (0.15&)	Heart failure, High cholesterol, Hypertension, Ischemic heart disease	11 (0.22%)	Heart failure, High cholesterol, Hypertension, Ischemic heart disease	2833 (0.31%)	Depression, Diabetes II, High cholesterol, Hypertension	974 (0.41%)
5	High cholesterol, Hypertension, Ischemic heart disease, Peripheral artery occlusive disease	32 (0.13%)	High cholesterol, Hypertension, Ischemic heart disease, Osteoarthritis	10 (0.20%)	Depression, Diabetes II, High cholesterol, Hypertension	2337 (0.25%)	Atrial fibrillation, High cholesterol, Hypertension, Ischemic heart disease	893 (0.38%)

Each chronic condition portfolio in this table represents individuals who have exactly that specific combination of conditions and no others. The risk factors are included in this table

### Sensitivity analysis

**Fig. 3:**
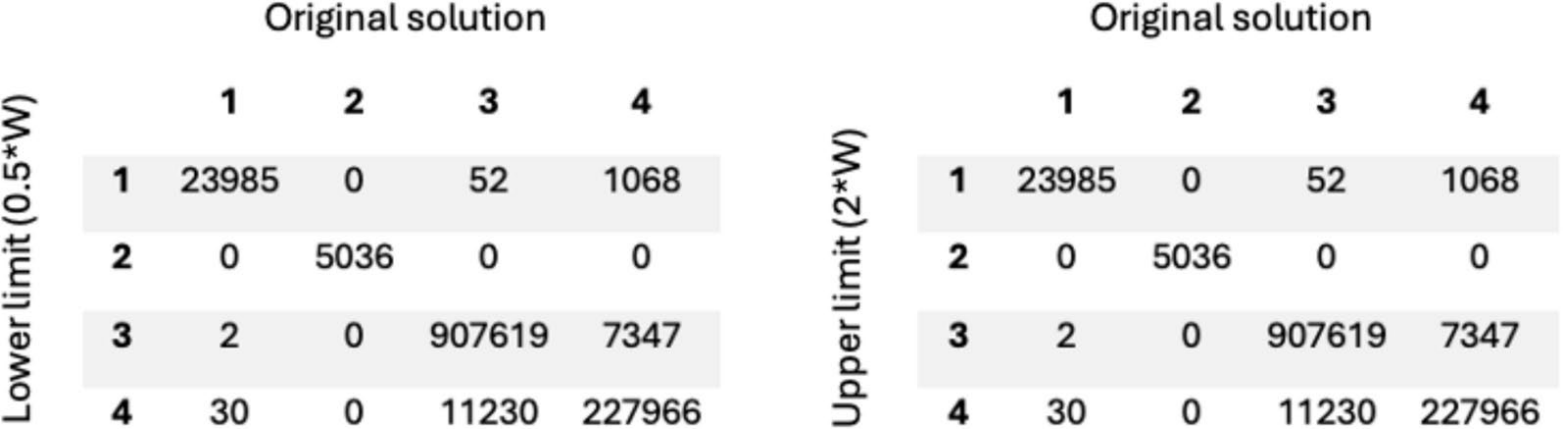
Illustrates two confusion matrices. The left compares the original solution with the lower limit (0.5*W), while the one on the right compares the original solution with the upper limit (2*W)

To assess the robustness and stability of the clustering solution, a sensitivity analysis was performed. This investigated whether the results were sensitive to the relationship between chronic conditions and healthcare utilization. In this analysis, the weight between the two data segments, chronic conditions and healthcare utilization, was adjusted. This allowed healthcare utilization to be weighted more or less in the clustering process, relative to the chronic conditions. The lower limit was set to 0.5*W and the upper limit to 2*W, where W is the weight. This range was chosen to reflect meaningful but realistic perturbations, with the aim of assessing robustness of the clustering solution rather than optimizing the weighting scheme. Figure 3 illustrates the weight adjustments and their impact on cluster formation through confusion matrices.

As shown in Fig. 3, two confusion matrices were presented, comparing the original solution to the lower limit (0.5*W) and the upper limit (2*W), respectively. The two matrices were identical, as both limits resulted in the same clustering solution. In both matrices, most observations were located along the diagonal (98.3%), indicating a general similarity between the cluster assignments. Notable deviations were observed in clusters 3 and 4, minor discrepancies in cluster 1, and complete consistency in cluster 2. The results indicated that the clustering solution is reasonably stable within the examined range of weight, with only minor deviations observed.

## Discussion

### Summary of main findings

The aim of this study was to group patients with multimorbidity based on their chronic conditions and healthcare utilization in order to identify relevant patterns, especially among those with high healthcare utilization. The study was based on a multimorbid population of 1,184,334 individuals with two or more of the chronic conditions across all age groups listed in Table 1. The average age in the population was 65 years, and the proportion of women was slightly higher (54.5%). On average, individuals had 3.26 chronic conditions. Based on this cohort, four clusters were identified using a weighted k-means clustering approach. The four clusters, which were formed based on chronic conditions and healthcare utilization variables, were further described using sociodemographic variables.

### Characteristics of clusters

Cluster 1 stood out from the other clusters by having the highest healthcare utilization, including hospital admissions (4.2), bed days (29.5), medication use (9.3), and outpatient visits (16.1). A possible explanation for this high level of use could be the greater disease burden, reflected in the average number of chronic conditions (4.5) and the composition of chronic conditions. The elevated prevalence of kidney disease in this cluster may contribute to the high healthcare utilization, as patients receiving dialysis require frequent medical attention and are often severely ill [29–31]. Mental illnesses were highly overrepresented in this cluster, especially severe disorders such as schizophrenia, personality disorders, and addictive disorders, which are often associated with complex treatment needs and low levels of self-management [32, 33]. Moreover, the combination of somatic and mental health problems was known to increase the risk of complications and reduce treatment effectiveness, which may have further contributed to the high healthcare utilization [34]. The combination of low education, low income, and a high proportion of individuals living alone may also have led to poorer health outcomes and created challenges in navigating the healthcare system and managing care independently [35–37]. Therefore, it was likely that both condition-related and social factors played a key role in the high healthcare utilization observed in this cluster.

Cluster 2 was the smallest cluster and was characterized by a relatively young population (mean age 51.5 years) and a high proportion of women (75.6%). The cluster was characterized by a relatively low average number of chronic conditions (2.98), but a high prevalence of mental disorders such as depression, anxiety, and personality disorders. This was reflected in healthcare utilization, where the number of psychologist consultations was remarkably higher than in the other clusters. In addition, this cluster showed high levels of education, income, and employment, which may have made it easier to access healthcare services, especially psychological treatment, where personal financial contribution is often a barrier [35–37]. The high number of psychologist visits could therefore reflect both a high need and better opportunities for accessing care. Despite having relative few somatic chronic conditions, this cluster showed that mental health issues could lead to considerable healthcare utilization. This highlighted the importance of including mental conditions in the understanding of disease burden and healthcare use, even among younger and socioeconomically advantaged individuals.

Cluster 3 represented the majority of the multimorbidity population (77.6%) and was characterized by the lowest use of healthcare utilization. This cluster also had the second-lowest average number of chronic conditions (3.08) and was mainly characterized by chronic conditions that are often easier to manage, such as hypertension, high cholesterol, and osteoarthritis. Additionally, the cluster showed a relatively high social position, with the second-highest levels of education and income, and many individuals were still employed. These factors may have supported better self-care and disease management, which could have contributed to the low healthcare utilization.

Cluster 4 was characterized by the highest average age (69.2 years) and the second highest number of average chronic conditions (3.85). This cluster stood out due to its high healthcare utilization, particularly visits to general practitioners and specialists. This may be linked to both age and chronic condition patterns, as several of the overrepresented conditions, including atrial fibrillation, heart failure, and Parkinson’s disease, require close monitoring and follow-up in both primary and secondary care settings. The high number of visits to general practitioners may be related to the need for coordinating treatments across multiple conditions and healthcare providers. This may be due to that many individuals in this cluster had complex condition trajectories that required ongoing follow-up. Additionally, the high number of visits to specialists could indicate that many needed assessment and treatment for specialized health issues, possibly several at once. Despite the extensive contact with the healthcare system, the number of bed days and psychological visits was low. This could suggest that healthcare utilization was mainly driven by somatic conditions that did not require long-term hospitalization, rather than by mental health conditions.

### Comparison with previous studies

Previous studies have investigated multimorbidity patterns and their association with healthcare utilization. However, it should be noted that there are differences in definitions and methodological approaches when comparing results. In this study, the definition of multimorbidity included both somatic and mental chronic conditions, with mental disorders making a substantial contribution to healthcare utilization.

Ansari et al. [38] identified five multimorbidity clusters where complex multimorbidity and chronic condition combinations involving hypertension, gastrointestinal, and musculoskeletal conditions were associated with higher healthcare utilization [38]. Similar to our findings, they observed that individuals in more complex clusters, in terms of number of chronic conditions, had higher healthcare utilization. However, Ansari et al. [38] primarily focused on somatic chronic conditions and thereby potentially underestimating the full healthcare burden associated with the combination of mental and somatic conditions.

Juul-Larsen et al. [15] identified eight multimorbidity clusters among acutely hospitalized older patients in Denmark using latent class analysis (LCA). They found that combinations involving major chronic conditions such as diabetes and COPD were associated with increased healthcare utilization [15]. Stockmarr et al. [14], which in principle examined the same population but with different chronic conditions, identified five clusters using the k-means algorithm: allergies, chronic heart conditions, diabetes, hypercholesterolemia, and musculoskeletal and psychiatric conditions. The chronic heart conditions cluster was found to have the highest healthcare utilization [14].

Ansari et al. [38], Juul-Larsen et al. [15], and Stockmarr et al. [14] grouped individuals based solely on chronic condition patterns without standardizing the variables, meaning that more prevalent conditions had greater influence on the cluster formation. Furthermore, Juul-Larsen et al. [15] applied LCA, which is a probabilistic method, while this study applied a weighted k-means algorithm to standardized variables, thereby weighting all chronic conditions equally.

This difference in approach has important implications. By standardizing the variables, this study prevents highly prevalent chronic conditions from dominating the clustering solution, and thereby enables the identification of subgroups based on combinations of both common and rarer chronic conditions. While this reduces the epidemiological prevalence, it may provide a more pathologically oriented understanding of how chronic conditions co-occur. In contrast, non-standardized approaches offer a more epidemiological interpretation by emphasizing prevalent conditions. However, they may risk overlooking less common but clinically meaningful combinations of chronic conditions.

In addition, our applied weighting of healthcare utilization variables ensured a balanced influence between chronic conditions and healthcare utilization in the clustering process. This approach allowed the identification of clusters that not only differ in compositions of chronic conditions but also in healthcare utilization patterns associated with these combinations. Hence, the weighting contributes to an integrated understanding of multimorbidity patterns and their real-world impact on healthcare utilization. However, future work could explore data-driven or outcome-optimized weighting approaches, for example, using data-driven optimization approaches.

### Clinical and public health implications

Identification of different multimorbidity clusters highlights that patients with multimorbidity represent a heterogeneous group with varying needs. The findings from this study suggest that targeted interventions should be considered for specific clusters. For instance, for cluster 1, ensuring continued access to psychological support and close follow-up that addresses both somatic and mental health conditions may be essential. For cluster 2, enhanced mental health support may be needed for women with early multimorbidity. Cluster 3, which is identified as a large group with low healthcare utilization, may benefit from preventive efforts to avoid future deterioration. Finally, for cluster 4, coordinated somatic care may be more appropriate for older individuals with complex chronic condition patterns.

Knowledge from the cluster analysis can support more efficient resource allocation and serve as a basis for the development of differentiated care pathways that better match patients’ needs. In addition, it may help healthcare professionals in the early identification of patients with potentially high care needs, allowing for adjusted follow-up and support based on the characteristics of each cluster. In clinical practice, the use of cluster information can aid in risk assessment, resource prioritization, and adjustment of treatment strategies.

### Methodological considerations and limitations

This study is based on register data that includes information on chronic conditions, medication use, healthcare utilization, and sociodemographic factors. However, a limitation of this study is the absence of lifestyle and functional status variables, such as smoking, alcohol consumption, and physical activity, which were not available in the nationwide registry data. These factors are known to influence both the development of chronic conditions and healthcare utilization and could therefore contribute to unmeasured differences between clusters. Their omission may result in residual confounding, where part of the variation in hospitalization patterns reflects underlying lifestyle-related differences rather than disease combinations alone. Another limitation is the inclusion of hypertension, high cholesterol, and obesity as chronic conditions rather than as risk factor in the clustering process. Although these conditions are often precursors to other chronic conditions, they were included in line with previous multimorbidity studies [24, 25] and due to their high prevalence, chronic nature, and substantial contribution to healthcare utilization. Nevertheless, their inclusion may have amplified their influence on the clustering structure. This was addressed by excluding them in some of the later analyses, such as the chronic condition portfolios in Appendix 1. Finally, the findings are based on Danish registry data, which may limit generalizability to other healthcare systems with different coding practices and population characteristics.

K-means clustering is not designed to yield equally sized clusters, and the dominance of one large cluster therefore reflects the population’s underlying multimorbidity structure rather than a methodological limitation. A large cluster can be interpreted as a baseline or common multimorbidity profile, while smaller clusters highlight more specific and clinically distinct subgroups.

Furthermore, K-means is a simple and effective method, but it requires that the number of clusters are determined. Alternative methods, such as DBSCAN, identify clusters based on density, using hyperparameters to control the sensitivity of cluster detection. While this removes the need to manually decide the numbers of clusters, DBSCAN is highly sensitive to these hyperparameters, and different settings can lead to substantially different results. [39]. Moreover, DBSCAN classifies observations that do not clearly belong to any cluster as outliers. Although, it is possible to assign these outliers to a separate cluster post hoc, this approach is not desirable, as it would result in an extremely heterogeneous group with limited clinical interpretability. Interpretation of DBSCAN clusters can also be more difficult, as they do not guarantee similarity among all points within the same cluster. Another alternative is fuzzy k-means, where observations are not assigned to a single cluster but instead have degrees of membership across multiple clusters [40]. This approach may be useful in situations with heterogeneous data, where strict cluster boundaries are less appropriate and probabilistic assumptions are undesirable. Other soft clustering methods, such as latent class analysis (LCA), also produce membership degrees, but the standard implementation of LCA typically assumes binary or categorical data and is therefore less suitable when working with standardized, continuous variables, such as healthcare utilization variables in this study [41]. However, the clinical relevance of soft clustering methods is debatable, as healthcare decisions often depend on clearly defined patient groups.

### Perspectives and future research

The current cluster analysis provides a valuable overview of multimorbidity and healthcare utilization in a large population. However, it also highlights several opportunities for future research. Since this study is based on a cross-sectional cohort, a different approach would be to include a longitudinal perspective. This could be approached in two ways: one possibility is to define clusters at a baseline and then examine how individuals within each cluster evolve over time regarding disease burden and healthcare utilization. This would allow for analysis of progression and outcomes while assuming that the baseline clusters remain fixed. Another approach is to apply clustering at multiple time points to detect changes in cluster structure over time, as suggested by Vetrano et al. [42]. In this case, new clusters would be identified at each time point, and individuals’ transitions between clusters could be analyzed. This approach would make it possible to examine how patterns of disease burden and healthcare utilization change over time, including identification of trajectories associated with health deterioration or increased service needs.

Future studies could also benefit from including additional variables such as functional ability, quality of life, and social networks. These factors may offer a more nuanced understanding of the different clusters and provide a broader picture of individuals’ overall needs and resources. This could enhance the clinical relevance of the results.

The findings from this study also have potential for use in predictive models. For example, specific clusters, such as those with the highest hospitalization, could be used as a starting point to develop models that identify patients at high risk of frequent hospital admissions. Overall, the study suggests several paths for future research that could strengthen the practical application of the results and contribute to a deeper understanding of the complexity of multimorbidity.

## Conclusion

This study applied a weighted k-means method to identify four clusters in the entire population of individuals with multimorbidity in Denmark in 2019, defined by 33 chronic conditions. The clustering process included both chronic conditions and healthcare utilization variables. The clusters varied in chronic condition patterns and healthcare utilization, suggesting that tailored treatment approaches may be needed to address the specific needs of each patient group. Additionally, the results indicated that combinations of chronic conditions involving mental chronic conditions particularly contribute to high healthcare utilization, underscoring the importance of healthcare strategies that consider the interplay between mental and somatic conditions to better manage complex patients. Future research should explore how multimorbidity clusters can be used to strengthen prevention and treatment strategies, and whether specific chronic condition patterns or patient characteristics can predict increased healthcare utilization among multimorbid patients.

## Data Availability

Data that support the findings of this study are available from Statistics Denmark, but restrictions apply to their availability. The data were used under license for the current study and are not publicly available.

## Appendix

**Table 5:** presents the most prevalent combinations of two, three, and four chronic conditions (chronic condition portfolios) found within each cluster

2 conditions rank	Cluster 1	Cluster 1 Prevalence	Cluster 2	Cluster 2 Prevalence	Cluster 3	Cluster 3 Prevalence	Cluster 4	Cluster 4 Prevalence
1	Depression, Epilepsy	318 (1.33%)	Allergies, Depression	229 (4.55%)	Allergies, COPD	26,586 (2.89%)	Allergies, COPD	4332 (1.83%)
2	Depression, Stroke	121 (0.50%)	Depression, Epilepsy	175 (3.48%)	Osteoarthritis, Osteoporosis	10,695 (1.16%)	Diabetes 2, Osteoarthritis	2875 (1.22%)
3	Depression, Osteoarthritis	119 (0.50%)	COPD, Depression	157 (3.12%)	Depression, Osteoarthritis	10,473 (1.14%)	Depression, Osteoarthritis	2412 (1.02%)
4	Anxiety, Depression	117 (0.49%)	Depression, Osteoarthritis	148 (2.94%)	Depression, Epilepsy	8970 (0.98%)	Osteoarthritis, Osteoporosis	2355 (1.00%)
5	COPD, Depression	116 (0.48%)	Allergies, COPD	147 (2.92%)	Diabetes II, Osteoarthritis	8148 (0.89%)	Depression, Diabetes II	2008 (0.85%)
3 conditions rank	Cluster 1	Cluster 1 Prevalence	Cluster 2	Cluster 2 Prevalence	Cluster 3	Cluster 3 Prevalence	Cluster 4	Cluster 4 Prevalence
1	Atrial fibrillation, Heart failure, Ischemic heart disease	61 (0.25%)	Allergies, Depression, Osteoarthritis	73 (1.45%)	Allergies, COPD, Depression	2083 (0.23%)	Allergies, COPD, Osteoarthritis	731 (0.31%)
2	Depression, Epilepsy, Schizophrenia	46 (0.19%)	COPD, Depression, Osteoarthritis	27 (0.54%)	Allergies, COPD, Osteoarthritis	2049 (0.22%)	Allergies, COPD, Depression	696 (0.29%)
3	COPD, Depression Osteoporosis	42 (0.18%)	COPD, Depression, Osteoarthritis	16 (0.32%)	Allergies, COPD, Osteoporosis	1547 (0.17%)	Atrial fibrillation, Heart failure, Ischemic heart disease	638 (0.27%)
4	Depression, Epilepsy, Stroke	41 (0.17%)	Depression, Epilepsy, Osteoarthritis	16 (0.32%)	Atrial fibrillation, Heart failure, Ischemic heart disease	1465 (0.16%)	Diabetes 2, Ischemic heart disease, Osteoarthritis	609 (0.26%)
5	Atrial fibrillation, Heart failure, Osteoarthritis	33 (0.14%)	COPD, Depression, Epilepsy	15 (0.30%)	Depression, Osteoarthritis, Osteoporosis	1423 (0.15%)	Allergies, COPD, Osteoporosis	608 (0.26%)
4 conditions rank	Cluster 1	Cluster 1 Prevalence	Cluster 2	Cluster 2 Prevalence	Cluster 3	Cluster 3 Prevalence	Cluster 4	Cluster 4 Prevalence
1	Atrial fibrillation, Diabetes II, Heart failure, Ischemic heart disease	20 (0.08%)	Allergies, COPD, Depression, Osteoarthritis	11 (0.22%)	Atrial fibrillation, Diabetes II, Heart failure, Ischemic heart disease	357 (0.04%)	Atrial fibrillation, Diabetes II, Heart failure, Ischemic heart disease	225 (0.10%)
2	Atrial fibrillation, COPD, Heart failure, Ischemic heart disease	19 (0.08%)	COPD, Depression, Ischemic heart disease, Osteoarthritis	5 (0.10%)	Atrial fibrillation, Heart failure, Ischemic heart disease, Osteoarthritis	342 (0.04%)	Atrial fibrillation, Heart failure, Ischemic heart disease, Osteoarthritis	207 (0.09%)
3	Atrial fibrillation, Heart failure, Ischemic heart disease, Kidney disease	17 (0,07%)	COPD, Depression, Osteoarthritis, Osteoporosis	5 (0.10%)	Allergies, COPD, Osteoarthritis, Osteoporosis	326 (0.04%)	Allergies, COPD, Depression, Osteoarthritis	183 (0.08%)
4	Atrial fibrillation, Heart failure, Ischemic heart disease, Osteoarthritis	14 (0.06%)	Allergies, Bowel disease, Depression, Osteoarthritis	4 (0.08%)	Allergies, COPD, Depression, Osteoporosis	323 (0.04%)	Allergies, COPD, Osteoarthritis, Osteoporosis	178 (0.08%)
5	Atrial fibrillation, Heart failure, Ischemic heart disease, Stroke	14 (0.06%)	Allergies, COPD, Depression, Osteoporosis	4 (0.08%)	COPD, Depression, Osteoarthritis, Osteoporosis	265 (0.03%)	COPD, Depression, Osteoarthritis, Osteoporosis	169 (0.07%)

Each chronic condition portfolio represents individuals who have exactly that specific combination of conditions and no others. The risk factors are excluded from this table

## Code availability

Custom code is restricted to data protection regulations at Statistics Denmark.
